# Influence of Annealing on the Damping Behavior of Ni-Cu-Mn-Ga Ferromagnetic Shape Memory Alloys

**DOI:** 10.3390/ma13020480

**Published:** 2020-01-19

**Authors:** Xiaoqi Liao, Xin Xu, Lumei Gao, Muhammad Tahir Khan, Chunxi Hao, Fei Cheng, Yuewei He, Yu Wang

**Affiliations:** 1 MOE Key Laboratory for Nonequilibrium Synthesis and Modulation of Condensed Matter and State Key Laboratory for Mechanical Behavior of Materials, Xi’an Jiaotong University, Xi’an 710049, China; theliaospc@126.com (X.L.); haocx.edu.cn@stu.xjtu.edu.cn (C.H.); F_cheng1994@163.com (F.C.); h2693017403@stu.xjtu.edu.cn (Y.H.); 2 Instrument Analysis Center, Xi’an Jiaotong University, Xi’an 710049, China; lmgao@xjtu.edu.cn; 3 Faculty of Engineering and Applied Sciences, Department of Physics, RIPHAH International University I-14 Campus Islamabad, Islamabad 44000, Pakistan; Tahir_iiui14@yahoo.com

**Keywords:** ferromagnetic shape memory alloys, martensite, damping, twin boundary, atomic order

## Abstract

Damping materials have attracted much attention for wide potential applications in the industry. Previous research shows that annealing treatment is an effective and costless way of improving the functional properties of conventional shape memory alloys. However, there are few investigations concerning the annealing effect of the ambient-temperature damping behavior. In this paper, we present the influence of annealing treatment on the martensitic transformation and damping behaviors of Ni${}_{55-x}$Cu${}_{x}$Mn${}_{25}$Ga${}_{20}$ (*x* = 0, 2, 4, 6) alloys within the ambient-temperature range. With increasing annealing time, the martensitic transformation temperature and the temperature span of martensitic transformation decrease. Moreover, annealing treatment greatly enhances the twin boundary damping peak of martensite. The X-ray diffraction (XRD) measurement demonstrates that annealing can improve the degree of L2${}_{1}$ atomic order, which relieves the pinning effects for the twin boundary motion and thus leads to the enhancement of the twin boundary damping of these alloys.

## 1. Introduction

Damping materials designed for effectively suppressing undesirable noise and mechanical vibrations play an increasingly important role in the fields of high-speed vehicles and high-precision instruments [1]. The damping capacity (Q${}^{-1}$) or internal friction (IF) is used to represent the capacity of converting the mechanical energy into dissipating heat for damping materials. Numerous efforts have been continuously devoted to developing high damping materials over decades. Various damping mechanisms were reported, such as the movement of crystalline defects (point defects [2,3], dislocations [4], etc.) and the motion of planar interfaces (grain boundaries, phase interface, twin boundary, etc.) [5]. Therefore, the damping capacity of materials strongly relies on the behavior of mobile defects or interfaces.

Shape memory alloys (SMAs) are considered one of the most promising candidates for high damping materials, and damping behavior has been extensively studied in many alloy systems (Mn-Cu [6], Au-Cd [7], Ti-Ni [8], Ti-Ni-X (X = Cu, Fe) [9,10], Ti-Pd-X (X = Fe, Cu, Cr, Co) [11] and Ni-Mn-Ga [12,13,14,15,16,17,18,19,20,21]). The SMAs show two kinds of damping effects. The first one is a sharp damping peak depending on the temperature rate. It appears during the martensitic transformation regime and originates from the hysteretic movement of parent/martensite interfaces [11]. By contrast, the second one is independent on temperature rate and does not exhibit thermal hysteresis, which stems from the martensitic twin boundary (TB) motion under external stress [22,23,24,25,26]. From the perspective of practical application, the stable and broad TB damping peak is more desirable than the damping peak of martensitic transformation, which has become the key topic for most damping investigations.

Many SMAs such as Mn${}_{70}$Cu${}_{30}$ [6], Ti${}_{50}$Ni${}_{48}$Fe${}_{2}$ [10], Ti${}_{50}$Ni${}_{50}$ [27] and Ti${}_{50}$Ni${}_{30}$Cu${}_{20}$ [28] alloys have TB damping. However, the TB damping of these SMAs appears below 260 K, which is not suitable for the practical applications in ambient temperature. Thus, the SMAs with ambient-temperature TB damping attract more and more attentions recently. Because the TB damping of martensite occurs below $M_{S}$, the $M_{S}$ of SMAs should be well above 300 K to obtain the ambient-temperature TB damping. It was reported that the Ti${}_{50}$Pd${}_{45}$Cr${}_{5}$ and Ti${}_{50}$Pd${}_{38}$Co${}_{12}$ alloys have high $M_{S}$ (> 450 K) and exhibit the high TB damping covering the ambient-temperature range but the high price of them limits their applications. The Ni-Mn-Ga based ferromagnetic shape memory alloys (FSMAs) with the cheap price and high $M_{S}$ become a promising candidate to obtain the TB damping within the ambient-temperature range. Interestingly, the recent work does show that a high damping peak ∼0.08 with a wide ambient-temperature range (173 ∼ 425 K) was observed in Ni-Cu-Mn-Ga alloys [29]. It is well known that the annealing treatment not only poses a significant effect on the martensitic transformation [30,31] but also significantly improves the functional properties including magnetocaloric effect [32,33] and elastocaloric effect [34] of Ni-Mn based FSMAs, because the atomic order of these alloys can be modified by proper annealing [30,31,34]. Thus, it is interesting to study the influences of annealing treatment on the ambient temperature damping behavior of Ni-Mn based FSMAs.

In this work, we investigate the effect of annealing on the transforming and damping behavior of Ni${}_{55-x}$Cu${}_{x}$Mn${}_{25}$Ga${}_{20}$ (*x* = 0, 2, 4, 6) FSMAs within the ambient-temperature range. It was found that martensitic temperatures of the samples decrease and their TB damping peak temperatures increase with increasing annealing time. More importantly, annealing treatment can dramatically enhance the TB damping capacity. The XRD measurement reveals that annealing treatment improves the degree of L2${}_{1}$ atomic order and does not generate any new phases or precipitates. Previous investigations [30,31] show that substitutional defects (i.e., Mn (Ga) atoms occupying the Ga (Mn) sublattice) formed by quenching can go back to their own regular sublattice after annealing. The pinning effect for the twin boundary motion is significantly relieved due to the improvement of the atomic order, which is proposed to result in the improvement of TB damping capacity of the alloys. Our study demonstrates that annealing treatment is an effective way to enhance the TB damping behavior of Ni-Cu-Mn-Ga alloys.

## 2. Materials and Methods

The polycrystalline alloy samples with the nominal compositions of Ni${}_{55-x}$Cu${}_{x}$Mn${}_{25}$Ga${}_{20}$ (*x* = 0, 2, 4, 6) were prepared. Corresponding ingots were fabricated by arc melting Ni, Cu, Mn and Ga with 99.9% purity under argon atmosphere. The as-cast ingots were annealed at 1173 K for 24 h in evacuated quartz tubes and subsequently quenched into room temperature water. In order to investigate the effects of annealing on damping, the water quenched specimens were further annealed at 773 K for 5/10/20 h in evacuated quartz tubes and then cooled to room temperature in the furnace, because such a heat treatment procedure was used to tune the atomic order in Ni-Mn based FSMAs [32]. The martensitic transformation temperatures of the samples were determined by a differential scanning calorimetry (DSC, Q2000 from TA Instruments) with a temperature sweeping rate of 10 K/min. The B2–L2${}_{1}$ transition temperatures were characterized by differential thermal analysis (DTA, STA 449C from NETZSCH) with a temperature sweeping rate of 10 K/min. Bar-shaped specimens with the dimension of 15 × 2.5 × 2 mm${}^{3}$ were cut by a spark machine for dynamic mechanical analysis, which was performed in a dynamic mechanical analyzer (DMA, Q800 from TA Instruments) with a single cantilever clamp. The internal friction and storage modulus were measured at the step cooling procedure with the frequency of 0.4 Hz and the oscillation amplitude of 5 μm. During the step cooling procedure, the samples were initially set to the target testing temperature and then kept isothermally for 5 min to reach temperature equilibrium. The structure of the high temperature L2${}_{1}$ parent phase of the samples was monitored by X-ray diffraction (XRD-7000, Shimadzu). The powder samples were used for the X-ray diffraction (XRD) measurement.

## 3. Results and Discussion

**Figure 1:**
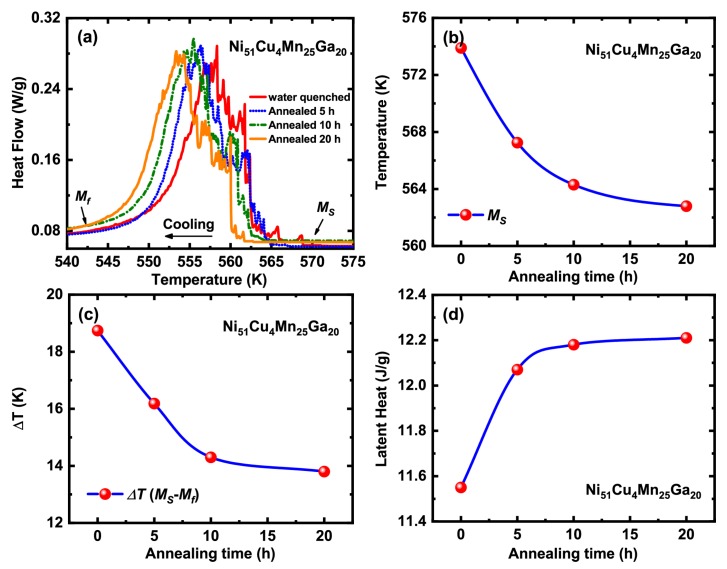
(**a**) Differential scanning calorimetry (DSC) curves of water quenched and annealed Ni${}_{51}$Cu${}_{4}$Mn${}_{25}$Ga${}_{20}$ alloys measured at 10 K/min upon cooling. (**b**) Annealing time dependence of the start martensitic transformation temperature ($M_{S}$). (**c**) The temperature span of martensitic transformation (ΔT) between $M_{S}$ and $M_{f}$ as a function of annealing time. (**d**) The transition latent heat as a function of annealing time.

Figure 1a shows the DSC curves of quenched and annealed Ni${}_{51}$Cu${}_{4}$Mn${}_{25}$Ga${}_{20}$ samples, which were measured upon cooling. The observed large exothermic peak is the signature for its martensitic transformations from the parent phase to the non-modulated tetragonal martensite [29]. These DSC peaks exhibit quite different characteristics as the annealing time extends. The starting temperature of martensitic transformation ($M_{S}$) decreases from 574 K to 563 K with the extension of annealing time, as displayed in Figure 1b. This demonstrates the annealing treatment promotes the phase stability of the austenite. Annealing also decreases the temperature span of martensitic transformation, which is equal to the temperature difference ΔT between $M_{S}$ and the finishing temperature of martensitic transformation ($M_{f}$) as shown in Figure 1c. Moreover, Figure 1d reveals that the latent heat of martensitic transformation obtained by integrating the corresponding exothermic peak becomes larger with prolonging annealing time. This manifests that the martensitic transformation becomes more dramatic after annealing treatment because certain disorder can be removed during the annealing process [30,31]. It is worth noting that the jerky characteristic of DSC peak during martensitic transformations was observed in both quenched and annealed samples. The jerky DSC peak is well observed in the Ni-Mn-Ga based FSMAs, which is associated with the interaction between the disorder (dislocations, local composition, vacancies, etc.) and austenite/martensite phase boundaries during the successive sudden progress of phase boundaries [35].

**Figure 2:**
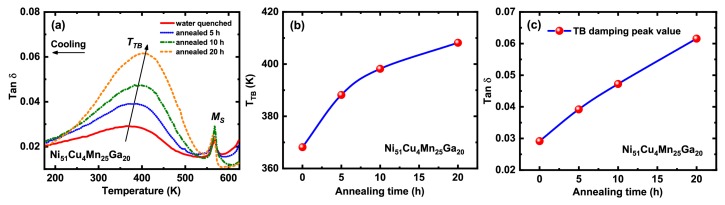
(**a**) Internal friction curves of water quenched and annealed Ni${}_{51}$Cu${}_{4}$Mn${}_{25}$Ga${}_{20}$ samples measured at the frequency of 0.4 Hz. (**b**) Twin boundary (TB) damping peak temperature ($T_{TB}$) as a function of annealing time. (**c**) Twin boundary damping peak value as a function of annealing time.

Figure 2a shows the internal friction versus temperature (tanδ–T) curves for the quenched and annealed Ni${}_{51}$Cu${}_{4}$Mn${}_{25}$Ga${}_{20}$ samples upon cooling. Two damping peaks were observed in their internal friction curves. The sharp damping peak located at high temperature is associated with martensitic transformation. The decrease of *M*${}_{S}$ with prolonging annealing time was also observed from the tanδ–T curves, which is consistent with data of DSC measurement. The broad damping peak at low temperature is related to the relaxational motion of a twin boundary [14], that is, the TB damping peak. The peak temperature of TB damping is defined as $T_{TB}$. With increasing annealing time, $T_{TB}$ shifts towards high temperature and an increment of 40 K is obtained for the 20-h annealed sample, which is displayed in Figure 2b. Meanwhile, a remarkable enhancement of TB damping capacity is observed in Figure 2c. The tanδ is enhanced by 117% after the 20-h annealing treatment. Thus, the data of Figure 2 demonstrates that the temperature distance between $M_{S}$ and $T_{TB}$ becomes smaller and TB damping capacity is improved by annealing.

**Figure 3:**
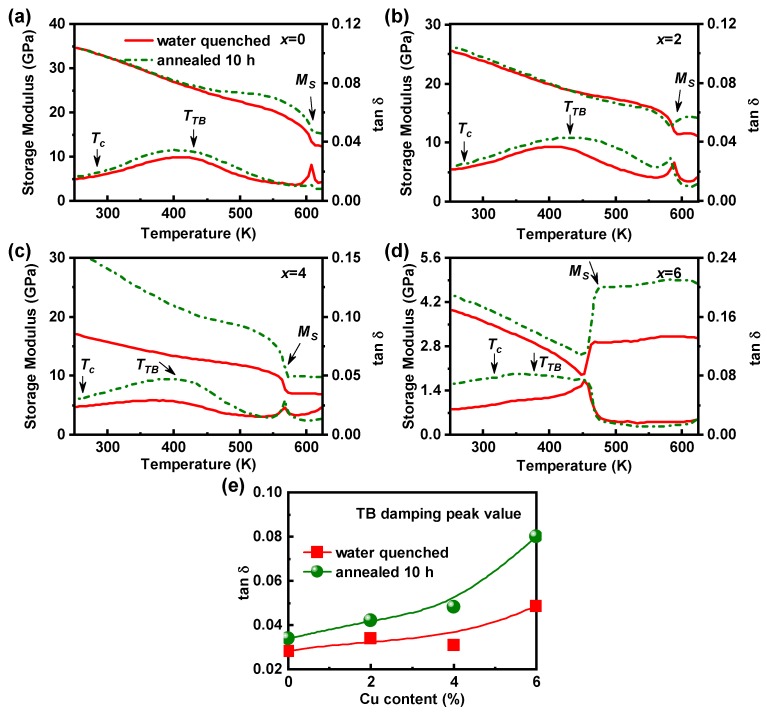
(**a**–**d**) Temperature dependence of storage modulus and internal friction for the water quenched and 10-h annealed Ni${}_{55-x}$Cu${}_{x}$Mn${}_{25}$Ga${}_{20}$ (*x* = 0, 2, 4, 6) alloys measured at 0.4 Hz. (**e**) Comparison of the TB damping peak values of water quenched and 10-h annealed Ni${}_{55-x}$Cu${}_{x}$Mn${}_{25}$Ga${}_{20}$ (*x* = 0, 2, 4, 6) samples.

To further clarify the influence of annealing treatment on the TB damping capacity of Ni${}_{55-x}$Cu${}_{x}$Mn${}_{25}$Ga${}_{20}$ (*x* = 0, 2, 4, 6) alloy system, their storage modulus and internal friction versus temperature curves under the condition of water quenching and 10-h annealing are compared in Figure 3a–d. It is obvious to see that TB damping capacity increases for all the samples after annealing. Moreover, Figure 3e reveals that the increment of TB damping capacity due to annealing becomes larger as the Cu content *x* increases. The Figure 3 well reveals that our annealing treatment method is effective to improve the TB damping of Ni-Cu-Mn-Ga FSMAs. The Curie temperatures ($T_{c}$) determined by Magnetization versus Temperature curves of 10-h annealed Ni${}_{55-x}$Cu${}_{x}$Mn${}_{25}$Ga${}_{20}$ (*x* = 0, 2, 4, 6) samples are 283 K, 271 K, 262 K and 316 K respectively [29], which are depicted in Figure 3a–d. $T_{c}$ of all these samples is lower than $T_{TB}$, proving that TB damping peaks are in paramagnetic state and no magnetic domains exist to affect their damping behavior. Moreover, the internal friction and storage modulus curves at/below $T_{c}$ do not show any anomalous, which suggests that magnetic domains have made an insignificant contribution to TB damping.

The substitution of Cu for Ni decreases the stability of the martensite, leading to the decrease of $M_{S}$ as shown in Figure 1b. Moreover, the distance between $M_{S}$ and $T_{TB}$ is reduced and the *p*-*d* orbital hybridization is weakened by the substitution of Cu for Ni, which results in the softening of the martensite modulus [29]. Therefore, the twin boundary mobility is enhanced by the modulus softening. This causes the increase of TB damping peak with Cu doping, as displayed in Figure 3e. It is worth to note that Cu doping produces similar effects on $M_{S}$ and TB damping for the water quenched and annealed samples.

The TB damping capacity is dependent on the twin boundary mobility [10]. It becomes larger as twin boundaries become more mobile. The twin boundary mobility is determined by two factors. One is the intrinsic twin boundary energy. It is proportional to $E\varepsilon^{2}$, where *E* is the modulus and ε is the twinning shear strain of martensite [36]. The other is the barrier energy due to the pinning effect of defects on twin boundaries. It is important to clarify which factor dominates the enhancement of TB damping by annealing.

**Figure 4:**
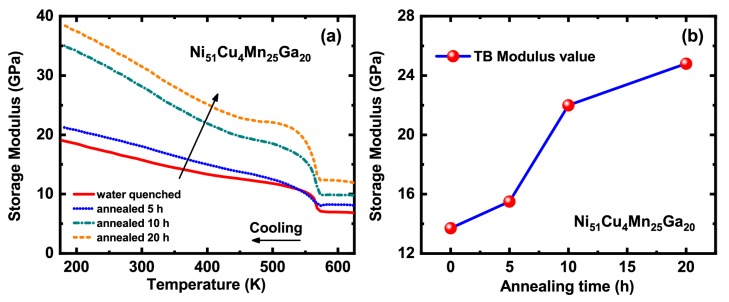
(**a**) Temperature dependence of storage modulus for the water quenched and annealed Ni${}_{51}$Cu${}_{4}$Mn${}_{25}$Ga${}_{20}$ alloys measured at the frequency of 0.4 Hz. (**b**) Storage modulus value corresponding to twin boundary damping peak as a function of annealing time.

To identify the change of intrinsic twin boundary energy after annealing, the temperature dependence of storage modulus of Ni${}_{51}$Cu${}_{4}$Mn${}_{25}$Ga${}_{20}$ sample for different annealing time (0/5/10/20 h) is compared in Figure 4a. The sharp storage modulus dip at high temperature corresponds to martensitic transformation. The storage modulus curve in martensitic state monotonously increases upon cooling and shows an anomalous around its corresponding damping peak temperature range. The storage modulus *E*’ gets larger with increasing the annealing time (Figure 4a), which can be seen more clearly from the change of martensitic storage modulus at $T_{TB}$ with the annealing time (Figure 4b). The shear strain of martensite ε is insensitive to annealing. Thus, such a hardening of martensite modulus increases the intrinsic twin boundary energy, which is suppose to hinder TB motion and reduce TB damping, being contradictory with the experimental observation in Figure 2a. Therefore, the increase of intrinsic twin boundary energy after annealing is not the dominant contribution to the enhancement of TB damping by annealing.

It is well known that Ni-Mn-Ga based FSMAs undergo an order-disorder transition from CsCl-type B2 structure with disordered atomic occupation into to the L2${}_{1}$ structure with next-nearest neighbor atomic ordering [30,37] at a very high temperature (950∼1050 K). However, water quenching from high temperature leads to a low degree of L2${}_{1}$ atomic order because there are many atoms occupying wrong sublattice sites, which are the substitutional point defects in the system. The atomic order of quenched alloys can be improved by proper annealing [30,31,32,33,34]. Therefore, to clarify the change of barrier energy of defect/twin boundary interaction after annealing, the influence of annealing on the L2${}_{1}$ atomic order of Ni${}_{51}$Cu${}_{4}$Mn${}_{25}$Ga${}_{20}$ is investigated.

**Figure 5:**
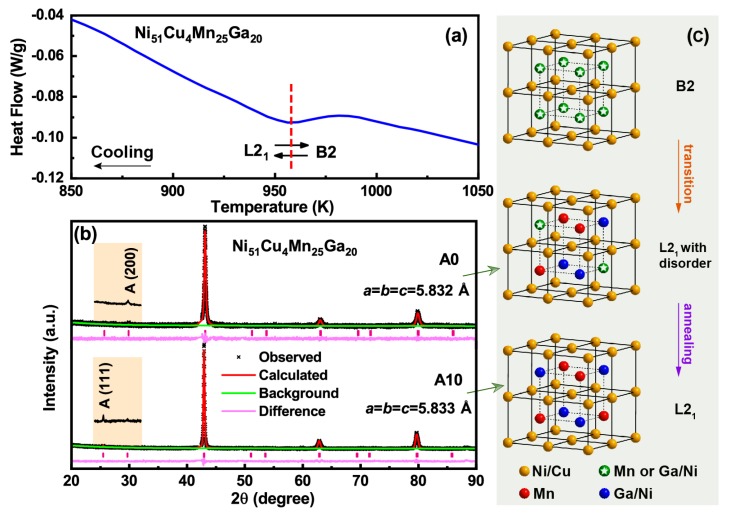
(**a**) Differential thermal analysis (DTA) curve of water quenched Ni${}_{51}$Cu${}_{4}$Mn${}_{25}$Ga${}_{20}$ alloy upon cooling. (**b**) X-ray diffraction (XRD) spectra of water quenched and 10-h annealed Ni${}_{51}$Cu${}_{4}$Mn${}_{25}$Ga${}_{20}$ alloys measured at 673 K. Insets show the XRD profile from 24${}^{\circ }$ to 32${}^{\circ }$. (**c**) A schematic illustration for the crystal structure evolution of atomic order degree after quenching and annealing treatment. Ni/Cu, Mn and Ga/Ni in different occupied sites are represented by yellow, red and blue spheres respectively. And the green balls with a pentagram indicates the random occupation of Mn and Ga/Ni atoms. (For interpretation of the references to color in this figure legend, the reader is referred to the web version of this article).

The DTA curve of water quenched Ni${}_{51}$Cu${}_{4}$Mn${}_{25}$Ga${}_{20}$ alloy in Figure 5a shows that the B2 to L2${}_{1}$ transition takes place around 960 K. Figure 5b shows the XRD spectra measured at 673 K for water quenched and 10-h annealed Ni${}_{51}$Cu${}_{4}$Mn${}_{25}$Ga${}_{20}$ alloys. Their corresponding refinement results were obtained from a least-squares fitting by using the GSAS Rietveld software [38,39]. The austenitic phase demonstrates a cubic L2${}_{1}$ structure for the two samples. The lattice parameters are fitted to be *a* = 5.8320 Å for the water quenched sample and *a* = 5.8331 Å for the 10-h annealed sample. Insets of Figure 5b show the XRD profile within the 2θ range of 24${}^{\circ }$ to 32${}^{\circ }$. In comparison with the water quenched sample, the (111) diffraction peak of 10-h annealed sample grows larger and its (200) diffraction peak almost disappears, demonstrating the L2${}_{1}$ atomic order is improved by annealing treatment [32]. The schematic illustrations of the atomic order after different treatment histories are revealed in Figure 5c. Above the atomic order-disorder transition temperature, the sample has a B2 structure, in which the Mn and Ga/Ni atoms occupy the sites randomly as indicated by the green balls with a pentagram. After rapid quenching from high temperature, the imperfect L2${}_{1}$ order and some substitutional defects can be kept. The number of defects reduces due to the increase of the degree of L2${}_{1}$ order after annealing.

Since the annealing treatment improves the degree of L2${}_{1}$ atomic order, the substitutional defects (Mn (Ga) atoms occupying the Ga (Mn) sublattice) formed by quenching can go back to their own regular sublattice. That is, some substitutional defects are eliminated after annealing. The pinning effect for the twin boundary motion is partially relieved. Thus, the barrier energy of defect/twin boundary interaction is reduced, which promotes the mobility of twin boundaries and its corresponding TB damping capacity. Considering that annealing treatment increases the intrinsic twin boundary energy and reduces TB damping as mentioned above, the reduction of barrier energy of defect/twin boundary interaction is the major contribution to the enhancement of TB damping after annealing.

It is interesting to note that the internal friction of austenite decreases (Figure 2a) and the corresponding storage modulus increases (Figure 4a) after annealing. This effect can also be understood by the annealing induced enhancement of atomic ordering. After annealing, fewer defects anticipate the migration in the austenite lattice, which results in the reduction of its damping capacity. For the same reason, fewer defects can response to the external stress in the austenite, leading to the enhancement of its storage modulus. Moreover, the lowering of defects concertation also reduces the free energy of the parent phase, which makes the parent phase more stable and leads to the decrease of martensitic transformation temperature after annealing. The energy barrier of martensitic transformation also becomes smaller due to the lowering of defects concertation, which decreases the temperature span of martensitic transformation after annealing.

## 4. Conclusions

In summary, the effects of annealing on the damping capacity of Ni${}_{55-x}$Cu${}_{x}$Mn${}_{25}$Ga${}_{20}$ (*x* = 0, 2, 4, 6) alloys are investigated. The annealing treatment decreases the martensitic transformation temperature and the temperature span of the martensitic transformation. More importantly, we found that annealing treatment greatly enhances the TB damping peak of these alloys. It is proposed that the degree of L2${}_{1}$ order improves after annealing, which relieves the pinning effects for the twin boundary motion. This is the major contribution to the enhanced TB damping after annealing. Our finding demonstrates that the annealing treatment is very effective to tune the ambient-temperature TB damping of Ni-Mn-Ga based FSMAs.

## References

[1] Chung D.D.L. (2001). Materials for vibration damping. J. Mater. Sci..

[2] Snoek J.L. (1941). Effect of small quantities of carbon and nitrogen on the elastic and plastic properties of iron. Physica.

[3] Zener C. (1947). Stress induced preferential orientation of pairs of solute atoms in metallic solid solution. Phys. Rev..

[4] Batist D. (1972). Internal Friction of Structural Defects in Crystalline Solids.

[5] Schaller R., Fantozzi G., Gremaud G. (2001). Mechanical Spectroscopy Q^−1^ 2001.

[6] Yin F.X., Sakaguchi T., Tian Q.C., Sakurai A., Nagai K. (2005). The twinning microstructure and damping behavior in Mn-30Cu (at %) alloy. Mater. Trans..

[7] Fan G., Zhou Y., Otsuka K., Ren X., Suzuki T., Yin F. On the internal friction due to the twin boundary-H interaction in martensite. Proceedings of the International Conference on Martensitic Transformations (ICOMAT-08).

[8] Yoshida I., Ono T., Asai M. (2000). Internal friction of Ti-Ni alloys. J. Alloys Compd..

[9] Yoshida I., Monma D., Iino K., Ono T., Otsuka K., Asai M. (2004). Internal friction of Ti-Ni-Cu ternary shape memory alloys. Mater. Sci. Eng. A.

[10] Fan G.L., Zhou Y.M., Otsuka K., Ren X. (2006). Ultrahigh damping in R-phase state of Ti-Ni-Fe alloy. Appl. Phys. Lett..

[11] Xue D.Z., Zhou Y.M., Ding X.D., Otsuka K., Lookman T., Sun J., Ren X.B. (2015). Ambient-temperature high damping capacity in TiPd-based martensitic alloys. Mater. Sci. Eng. A.

[12] Wang W.H., Ren X., Wu G.H. (2006). Martensitic microstructure and its damping behavior in Ni_52_Mn_16_Fe_8_Ga_24_ single crystals. Phys. Rev. B.

[13] Seguí C., Cesari E., Pons J., Chernenko V. (2004). Internal friction behaviour of Ni-Mn-Ga. Mater. Sci. Eng. A.

[14] Chang S.H., Wu S.K. (2008). Low-frequency damping properties of near-stoichiometric Ni_2_MnGa shape memory alloys under isothermal conditions. Scr. Mater..

[15] Liu J.Y., Wang J.M., Jiang C.B., Xu H.B. (2013). Internal friction associated with the premartensitic transformation and twin boundary motion of Ni_50+x_Mn_25−x_Ga_25_ (*x*= 0 − 2) alloys. J. Appl. Phys..

[16] Aaltio I., Lahelin M., Söderberg O., Heczko O., Löfgren B., Ge Y., Seppälä J., Hannula S.P. (2008). Temperature dependence of the damping properties of Ni-Mn-Ga alloys. Mater. Sci. Eng. A.

[17] Aaltio I., Mohanchandra K.P., Heczko O., Lahelin M., Ge Y., Carman G.P., Söderberg O., Löfgren B., Seppälä J., Hannula S.P. (2008). Temperature dependence of mechanical damping in Ni-Mn-Ga austenite and non-modulated martensite. Scr. Mater..

[18] Gavriljuk V.G., Söderberg O., Bliznuk V.V., Glavatska N.I., Lindroos V.K. (2003). Martensitic transformations and mobility of twin boundaries in Ni_2_MnGa alloys studied by using internal friction. Scr. Mater..

[19] Wang W.H., Liu G.D., Wu G.H. (2006). Magnetically controlled high damping in ferromagnetic Ni_52_Mn_24_Ga_24_ single crystal. Appl. Phys. Lett..

[20] Chernenko V.A., Pons J., Seguí C., Cesari E. (2002). Premartensitic phenomena and other phase transformations in Ni-Mn-Ga alloys studied by dynamical mechanical analysis and electron diffraction. Acta Mater..

[21] Stuhr U., Vorderwisch P., Kokorin V.V., Lindgård P.A. (1997). Premartensitic phenomena in the ferro- and paramagnetic phases of Ni_2_MnGa. Phys. Rev. B.

[22] Otsuka K., Wayman C.M. (1999). Shape Memory Materials.

[23] Salje E.K.H., Zhang H., Idrissi H., Schryvers D., Carpenter M.A., Moya X., Planes A. (2009). Mechanical resonance of the austenite/martensite interface and the pinning of the martensitic microstructures by dislocations in Cu_74.08_Al_23.13_Be_2.79_. Phys. Rev. B.

[24] Ahadi A., Sun Q.P. (2013). Stress hysteresis and temperature dependence of phase transition stress in nanostructured NiTi-Effects of grain size. Appl. Phys. Lett..

[25] Šittner P., Novák V. (2000). Anisotropy of martensitic transformations in modeling of shape memory alloy polycrystals. Int. J. Plast..

[26] Cesari E., Seguí C., Pons J., Perelló F. (1996). Internal friction and Young modulus behaviour of hot-rolled Cu-Al-Ni-Ti shape memory alloys. J. Phys..

[27] Fan G.L., Otsuka K., Ren X.B., Yin F.X. (2008). Twofold role of dislocations in the relaxation behavior of Ti-Ni martensite. Acta Mater..

[28] Fan G., Zhou Y., Otsuka K., Ren X., Nakamura K., Ohba T., Suzuki T., Yoshida I., Yin F. (2006). Effects of frequency, composition, hydrogen and twin boundary density on the internal friction of Ti_50_Ni_50−x_Cu_x_ shape memory alloys. Acta Mater..

[29] Liao X.Q., Wang Y., Fan G.L., Liu E.K., Shang J.R., Yang S., Luo H.Z., Song X.P., Ren X.B., Otsuka K. (2017). High damping capacity of a Ni-Cu-Mn-Ga alloy in wide ambient-temperature range. J. Alloys Compd..

[30] Sánchez-Alarcos V., Recarte V., Pérez-Landazábal J.I., Cuello G.J. (2007). Correlation between atomic order and the characteristics of the structural and magnetic transformations in Ni-Mn-Ga shape memory alloys. Acta Mater..

[31] Recarte V., Pérez-Landazabal J.I., Sánchez-Alarcos V., Rodríguez-Velamazán J.A. (2012). Dependence of the martensitic transformation and magnetic transition on the atomic order in Ni-Mn-In metamagnetic shape memory alloys. Acta Mater..

[32] Ghosh A., Mandal K. (2014). Effect of structural disorder on the magnetocaloric properties of Ni-Mn-Sn alloy. Appl. Phys. Lett..

[33] Li S.D., Yuan Z.R., Lü L.Y., Liu M.M., Huang Z.G., Zhang F.M., Du Y.W. (2006). Effect of annealing on the magnetic entropy change of CoMnSb alloy. Mater. Sci. Eng. A.

[34] Huang C.H., Wang Y., Tang Z., Liao X.Q., Yang S., Song X.P. (2015). Influence of atomic ordering on elastocaloric and magnetocaloric effects of a Ni-Cu-Mn-Ga ferromagnetic shape memory alloy. J. Alloys Compd..

[35] Gallardo M.C., Manchado J., Romero F.J., del Cerro J., Salje E.K.H., Planes A., Vives E., Romero R., Stipcich M. (2010). Avalanche criticality in the martensitic transition of Cu_67.64_Zn_16.71_Al_15.65_ shape-memory alloy: A calorimetric and acoustic emission study. Phys. Rev. B.

[36] Diestel A., Backen A., Rößler U.K., Schultz L., Fähler S. (2011). Twin boundary energy of hierarchically twinned magnetic shape memory alloys. Appl. Phys. Lett..

[37] Overholser R.W., Wuttig M., Neumann D.A. (1999). Chemical ordering in Ni-Mn-Ga Heusler alloys. Scr. Mater..

[38] Toby B.H. (2001). EXPGUI, a graphical user interface for GSAS. J. Appl. Cryst..

[39] Larson A.C., Von Dreele R.B. (2004). General Structure Analysis System (GSAS).

